# Homeostatic maintenance via degradation and repair of elastic fibers under tension

**DOI:** 10.1038/srep27474

**Published:** 2016-06-09

**Authors:** Calebe Alves, Ascanio D. Araújo, Cláudio L. N. Oliveira, Jasmin Imsirovic, Erzsébet Bartolák-Suki, José S. Andrade, Béla Suki

**Affiliations:** 1 Universidade Federal do Ceará, Departamento de Física, Fortaleza, CE, 60451-970, Brazil; 2 Boston University, Department of Biomedical Engineering, Boston, MA, 02215, USA

## Abstract

Cellular maintenance of the extracellular matrix requires an effective regulation that balances enzymatic degradation with the repair of collagen fibrils and fibers. Here, we investigate the long-term maintenance of elastic fibers under tension combined with diffusion of general degradative and regenerative particles associated with digestion and repair processes. Computational results show that homeostatic fiber stiffness can be achieved by assuming that cells periodically probe fiber stiffness to adjust the production and release of degradative and regenerative particles. However, this mechanism is unable to maintain a homogeneous fiber. To account for axial homogeneity, we introduce a robust control mechanism that is locally governed by how the binding affinity of particles is modulated by mechanical forces applied to the ends of the fiber. This model predicts diameter variations along the fiber that are in agreement with the axial distribution of collagen fibril diameters obtained from scanning electron microscopic images of normal rat thoracic aorta. The model predictions match the experiments only when the applied force on the fiber is in the range where the variance of local stiffness along the fiber takes a minimum value. Our model thus predicts that the biophysical properties of the fibers play an important role in the long-term regulatory maintenance of these fibers.

The extracellular matrix (ECM) plays a role in tissue development, damage repair, diseases and aging by influencing cellular responses. For example, the stiffness of the substratum on which stem cells are cultured has been found to direct cellular differentiation^1^. On the other hand, cells sense the mechanical properties of the ECM as well as secrete ECM components^2^. This mutual dependence between the ECM and its embedded cells likely evolved soon after multicellular life emerged on Earth^3^.

Cellular maintenance of the ECM requires an effective regulation that balances enzymatic degradation with replacement of the digested fragments with newly synthesized molecules forming and shaping the fibrils and fibers of the ECM including collagen and elastin. In several organs and tissues such as the vasculature, skin, heart and periodontium, a quick turnover of collagen with half-lives between 20 and 250 days have been observed^4^,^5^. Additionally, the mechanical stresses imposed by exercise are known to induce rapid and significant collagen turnover within 72 hours even in tendon^6^, which has a very long turnover time of collagen^7^.

Despite the strong and constant cellular maintenance, the micro-structure of collagen appears to remain in a stable homeostatic state throughout most of adult life even when demanding mechanical stresses lead to incessant remodeling. For example, while the diameter distribution of collagen in mouse tail tendon undergoes major changes until the age of 3 months due to development, the distribution remains nearly independent of age between 4 and 23 months^8^, a range that spans the adult life of the mouse and corresponds to approximately 10 to 60 years of human life^9^. Similarly, collagen morphometry is also nearly constant throughout adult life in skin^10^. The functional consequence of the nearly constant diameter distribution is a stable strain energy density that maintains proper mechanical function and resistance to rupture during this time period^8^. These findings raise an important question in mechanobiology: “How are cells able to maintain such a homeostatic structure over a period that corresponds to decades of human life”? While several studies have reported on the details of short-term ECM maintenance^2^ and collagen biosynthesis^11^, much less is known about the truely long-term regulation of ECM composition and structure. It appears that some as yet unknown control mechanism regulates the maintenance of ECM structure throughout much of adult life until this mechanism eventually loses its efficiency due to aging as evidenced by the increasing irregularity of the collagen fiber structure^12^,^13^.

An important feature of the ECM is that its fibers are maintained under tension. Because tension is known to influence stiffness^14^, proteolytic degradation^15^ as well as fibril organization^16^, it is possible that the biophysical properties of the fibers play an important role in the long-term regulatory maintenance of these fibers. In a previous study^17^, we used a model to study particle diffusion, cleaving and the subsequent relaxation of fibers under tension. In the current study, we introduce a new statistical model of fibril maintenance that involves digestion and repair and incorporates cellular activity that depends on the stiffness of the fibrils. Comparison of the computational results with experimentally obtained collagen morphometry suggests that the long-term homeostasis of fibril structure requires interaction between the cells’ ability to measure fibril stiffness and the fibrils’ ability to alter its binding affinity through mechanical tension.

## Results

### Basic Model Formulation

**Figure 1:**
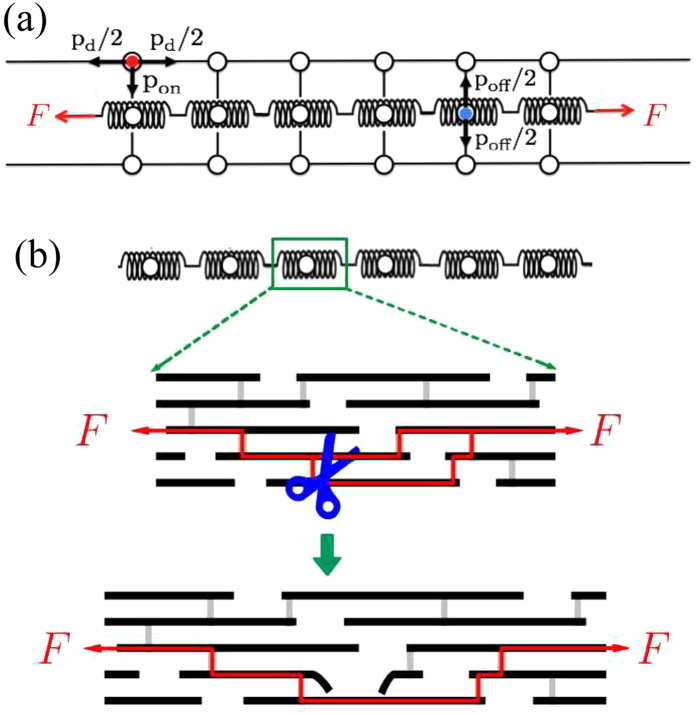
Schematic diagram of the computational model used in the simulations. (**a**) The chain of springs and binding sites are represented by small open circles. The two layers of sites surrounding the fiber are represented by the big open circles and the two types of particles are shown as filled circles with the red and blue circles corresponding to the degradative and regenerative particles, respectively. A particle at the bottom, can move up, left or right whereas a particle at the top can move down, left, or right. When a particle binds to a spring, it can stay there or move up or down. The ends of the chain are submitted to a constant force **F** during the entire simulation. A periodic boundary condition was applied along the *x* direction. (**b**) A zoom into one of the springs representing collagen monomers in parallel (black) reinforced by cross-links (gray). Red shows the force transmission pathways and the blue scissors represent a bound enzyme to a collagen monomer (middle diagram). Once the enzyme unbinds, the monomer is cleaved, but part of the monomer still participates in force transmission (bottom diagram).

To study how cells can balance enzymatic digestion and repair of a collagen fiber, we use a random walk model to mimic the diffusion along the fiber of two different types of molecular complex, which we refer to as regenerative (*R*) and degradative (*D*) particles. The *R* and *D* particles representing collagen monomers and collagen-cleaving enzymes such as matrix metalloproteinases, respectively, carry out the local fiber repair and digestion. The fiber consists of a one-dimensional chain of *N*_*s*_ linearly elastic springs in series (Fig. 1a). The fiber is surrounded by two layers of sites along which the *R* and *D* particles can diffuse and bind to the fiber at discrete locations corresponding to the springs. Periodic boundary conditions are applied in the *x* direction. Both ends of the chain are subject to a constant force that mimics tension on ECM fibers. To simulate the digestion and repair activity along the fiber, we begin with a chain having identical initial spring constants *k*(*t* = 0) ≡ *k*_0_.

The particle diffusion is initiated by releasing a set of particles of both types at random positions in the two layers surrounding the chain. The number of particles corresponding to the two types, *N*_R_ and *N*_D_, is initially fixed at time *t* = 0. Additionally, the particles do not interact with each other and there is no exclusion principle, which means that particles can simultaneously be at the same position surrounding the chain. However, only one particle is allowed to bind to any given binding site.

To carry out fiber maintenance, we apply the following rules to the diffusion and reaction processes depending on the type of particle:

1. The probability for a particle to move left or right along the chain is *p*_d_ and it is associated with diffusion;
2. The probability for a particle to move up from the bottom layer or move down from the top layer is *p*_on_. This step represents the event that a *D* or a *R* particle binds to the fiber. The specific rules that govern the binding processes depend on the model considered (see below) and the particle type. For *D* particles, the diffusion and binding processes are similar in the sense of probability resulting in an isotropic diffusion for all models considered. For *R* particles, the binding process depends on the specific model used and the process can be isotropic or anisotropic;
3. The probability for a bound particle of either type to leave the fiber and move up to the top layer or move down to the bottom layer is *p*_off_. This parameter controls the unbinding process and is associated with repair or degradation;

When a *D* or an *R* particle unbinds, the local spring constant *k* is reduced (*k* → *γk*) or increased (*k* → *k*/*γ*), respectively, by a constant factor *γ*. The reason for this is as follows. The fiber is composed of molecules in parallel and series. If the fiber was a regular array of molecules, cleavage would decrease the number of molecules in parallel. This would result in a linear decrease in the spring constant during subsequent cleavages and over a long time scale, it becomes possible that the local spring constant reaches zero effectively rupturing the chain. In reality, the molecules overlap and are connected via multiple inter-molecular as well as inter-fibril cross-links in a complex manner (Fig. 1b). Hence, the degradation of the fiber is also a complex process and a single cleaving event does not necessarily have to eliminate a complete monomer from the chain. Indeed, Fig. 1b demonstrates that following the cleavage of a monomer, part of the same monomer can still carry a force. Due to this complexity, simply subtracting one unit from *k* following an unbinding of a *D* particle is not appropriate. Therefore, we model the cleavage process by assuming that the decrease in spring constant is proportional to the actual value, which corresponds to a multiplicative degradation process. This assumes that the process of degradation is far away from the rupture threshold and this model provided an accurate description of stress relaxation and fragment release from elastin fibers^17^.

### Time Controlled Regulation of Fiber Maintenance

We consider two models with self-regulatory dynamics. In the first model, called the *temporal control model* (**TCM**), we incorporate the notion that cells are capable of actively measuring the stiffness of their surrounding matrix^18^,^19^ and respond by secreting degradative enzymes^20^ or upregulating enzyme inhibitors and eventually collagen production^21^. To mimic this behavior, the stiffness of the fiber is periodically evaluated at time intervals *t*_*u*_. If the stiffness is impaired (*K* < *K*_0_), a fraction *f* of the particles are randomly selected to be relabeled as *R* particles. Alternatively, if the stiffness is too high (*K* > *K*_0_), the same fraction *f* of the particles are randomly chosen to be relabeled as *D* particles. It is important to note that during this relabeling process the system does not recognize the particle type to be relabeled. However, statistically, this replacement process mimicking cellular response to alterations in fiber stiffness is able to change the overall concentration of particles around the fiber so as to regulate the overall maintenance process to be degradative or regenerative if the fiber is too stiff or too soft, respectively.

Table 1 summarizes the parameters of the model including those of the fiber.

It is possible to define the periodic control in many ways. For example, the stiffness of the fiber can be checked at every time step and relabeling particles can be based on whether *K* surpasses a predefined threshold below or above the target *K*_0_. We find, however, that such details about how the control is performed do not influence the general behavior of the system. Nevertheless, the presence of some periodic control is fundamental. If it was absent, it would be impossible for the fiber stiffness *K* to reach a steady state value, even if the number of particles of both types is always the same. The reason is that *K* is the harmonic mean of individual spring stiffnesses which introduces strong nonlinearity in the model with a tendency to decrease the overall fiber stiffness. Note, for example, that if one spring binds a *D* particle whereas another spring of the same stiffness binds an *R* particle, the two events do not compensate each other and the overall effect is to reduce *K*. To see this, consider the change in stiffness:


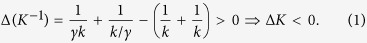


**Figure 2:**
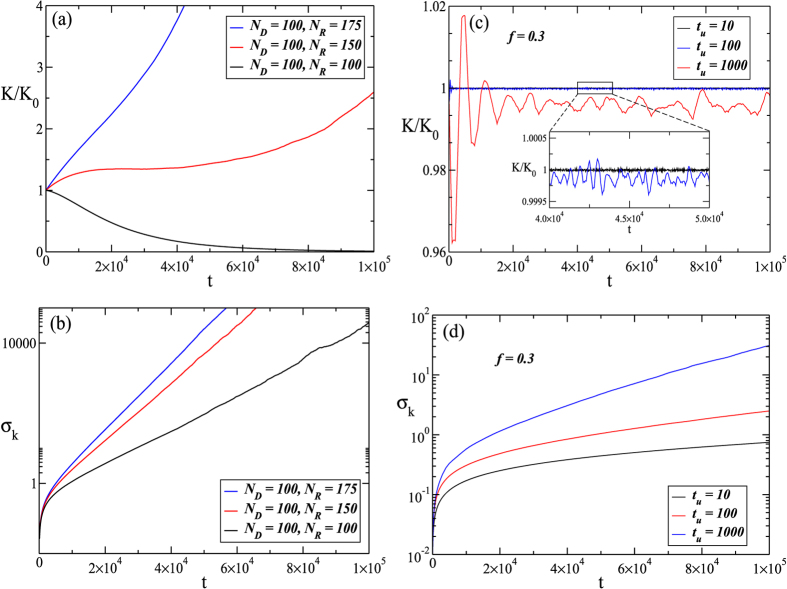
The normalized fiber stiffness and the standard deviation as a function of time. (**a**) The normalized stiffness *K*/*K*_0_, where *K*_0_ is the fiber stiffness at *t* = 0, as a function of time when no regulation is applied. The number of *D* and *R* particles are indicated by *N*_*D*_ and *N*_*R*_. (**b**) The standard deviation *σ*_*k*_ of the local spring constants *k* as a function of time when there is no regulation. In panels (c,d), we show the normalized stiffness and the standard deviation as a function of time for the **TCM** model. In both cases, colors indicate different time periods *t*_*u*_ at which the particles are updated to replace a fraction of them with new ones. The inset shows a close-up of the fiber stiffness for *t*_*u*_ = 10 and 100. The parameters used in the simulations were: *N*_*s*_ = 10^3^; *γ* = 0.995, *p*_*off*_ = 0.5. These results were obtained from an average of 500 simulations.

In order to maintain proper functionality, the cellular maintenance of a fiber needs to preserve the fiber’s elastic properties within a narrow target range. While a simple random walk model without control cannot achieve this, the **TCM** produces a stationary *K*. However, the **TCM** is unable to produce a homogeneous fiber since the standard deviation of fiber stiffness, *σ*_*k*_, diverges as shown in Fig. 2d. The reason for the divergence is the similarity of the model to a random walk. As the standard deviation of displacement for a simple random walker is proportional to *t*^*ν*^ with *ν* = 0.5, in our simulations the standard deviation for the distribution of *α* ~ log *k* varies according to *σ* ~ *t*^*ν*^ with *ν* ≈ 0.7. Here *α* is simply the difference between the number of visits of *D* and *R* particles a given spring has received. Supplementary Fig. S1 shows the distribution of *α* at five different time points.

### Spatio-temporal Control of Fiber Maintenance

To control both the mean and homogeneity of fiber stiffness, we introduce an additional feature in the model, the local control of the binding event based on the observation that the absence of tension on the ECM leads to disorganized fibers^16^. This new ingredient is expected to introduce an anisotropy in the diffusion-reaction process making the weaker springs more susceptible to be visited by *R* particles. We call this model *spatio-temporal control model* (**STCM**). While the behavior of the *D* particles remains the same as in the **TCM**, the probability *p*_*on*,*R*_ for an *R* particle to bind to the fiber now depends on the local stiffness with spring constant *k* as follows:


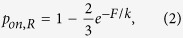


where the new parameter *F* is the force, per unit of length, applied to the ends of the chain. The force can be generated internally by contractile cells in the ECM^22^ or externally by for example blood pressure-related circumferential stress in the vessel wall^23^. Since *F* is the same at any location along the chain, a lower local value of *k* generates a higher local stretch. We assume that a higher local stretch unfolds cryptic binding sites^24^,^25^. Thus, depending on the local stiffness *k, p*_*on*,*R*_ can vary along the chain with higher values at regions where the fiber is weaker due to more frequent visits of *D* particles. Consequently, since *p*_*on*,*R*_ is higher at such locations, *R* particles tend to visit these sites with a higher probability. However, as soon as the *R* particles start to increase *k, p*_*on*,*R*_ naturally decreases recovering a value which corresponds to the isotropic diffusion-reaction case.

**Figure 3:**
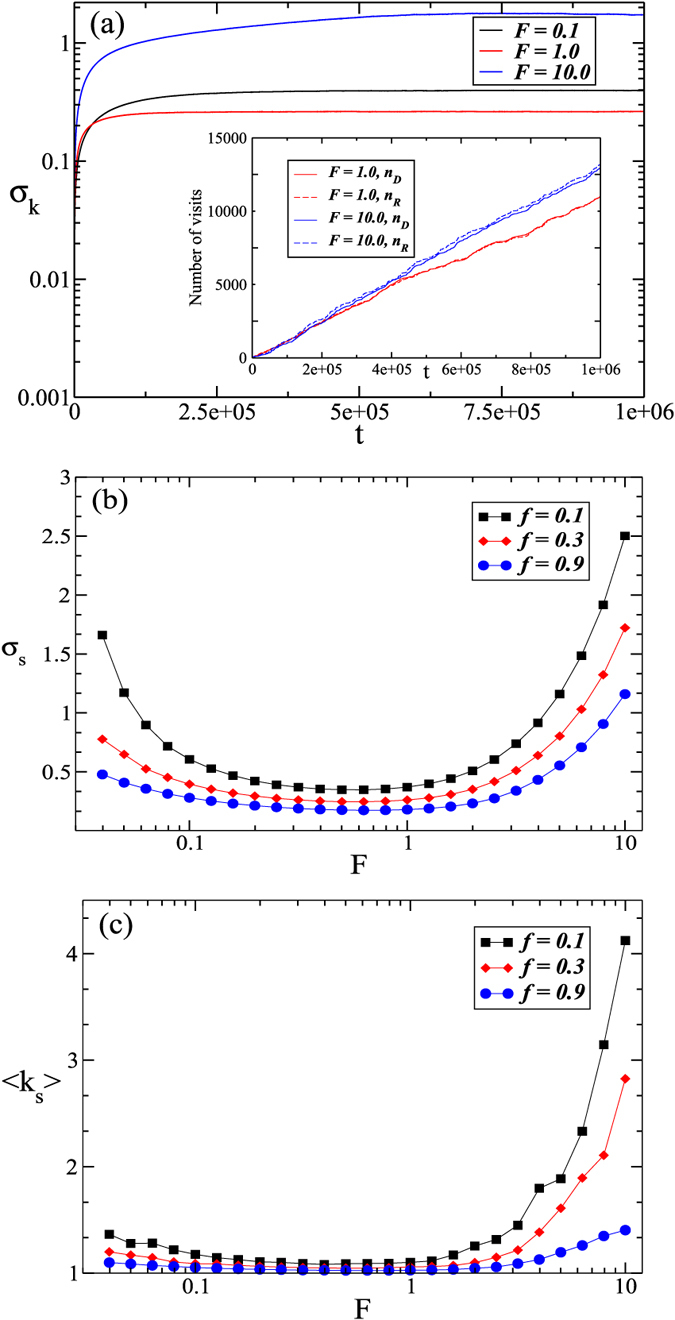
The mean and standard deviation of spring stiffnesses as a function of time for the STCM. In the main plot of (**a**), we show the standard deviation *σ*_*k*_ for three different applied forces *F*, when *t*_*u*_ = 10 and *f* = 0.3. For all three values of *F*, a steady state is reached after which *σ*_*k*_ displays only minor fluctuations. The inset shows *n*_*D*_ and *n*_*R*_, the number of visits of degradative and regenerative particles to an individual spring. Differences in the number of visits of both types is always greater for *F* = 10.0 than for *F* = 1.0. Panel 3b shows the standard deviation of springs stiffnesses after the steady state, *σ*_*s*_, is reached as a function of *F*. The curve reaches a minimum for *F* ≈ 0.63 for all three values of *f*. Panel 3c shows the mean spring stiffnesses after the steady state is reached as a function of *F*. The curve reaches a minimum for *F* ≈ 0.63 for all three values of *f*. These results were obtained from an average of 500 simulations.

During the digestion/repair processes *F* is assumed to be constant but *k* along the fiber shows substantial heterogeneity, as we have confirmed by the divergence in *σ*_*k*_ for both the random walk model without control and the **TCM**. To test whether the **STCM** can control the global elasticity while preserving fiber homogeneity, we carry out simulations with the **STCM** for different values of *f* and *t*_*u*_. We also examine the effect of *F* by varying it between 0.04 and 10. For all cases, the global fiber stiffness *K* is well controlled as in the **TCM**. However, *σ*_*k*_ no longer diverges. The main plot in Fig. 3a shows indeed that *σ*_*k*_ reaches a steady state although its value *σ*_*s*_ depends on *F*. Here, *σ*_*s*_ is the value around which *σ*_*k*_ fluctuates after it has reached a steady state.

Next, we run a set of simulations with various combinations of the parameters *F, t*_*u*_ and *f* in the **STCM**. For all parameter combinations, *σ*_*k*_ always reaches a steady state with *σ*_*s*_ depending on the actual parameters. We plot *σ*_*s*_ as a function of *F* for fixed *t*_*u*_ = 10 and three values of the fraction *f* in Fig. 3b. Surprisingly, *σ*_*s*_ presents a minimum as a function of *F* that occurs at *F* ≈ 0.63. The maximum homogeneity of stiffness along the fiber is thus achieved when *σ*_*s*_ is minimum which depends on the applied force as well as the strength of cellular control modeled by the fraction of particles *f* replaced during the fiber maintenance process. The decrease in *σ*_*s*_ for larger values of *f* results from the stronger and more effective feedback to regulate global stiffness through a higher concentration of control particles in the system. The presence of a minimum can be explained by recalling that the process of degradation and repair is similar to a random walk when *F* = 0 which results in a divergence of *σ*_*k*_. For any small positive value of *F*, the local control results in finite *σ*_*k*_ and with increasing *F*, the control becomes stronger and *σ*_*k*_ decreases. However, when *F* further increases, the control of the *R* particles becomes much faster than that of *D* particles leading to overshoots in repair and a subsequent increase in *σ*_*k*_ which we have verified by examining the local time variation of individual springs (not shown). Similarly to *σ*_*s*_, the mean fiber stiffness also shows a minimum for *F* ≈ 0.63 (Fig. 3c). For the strongest control when *f* = 0.9, the fiber stiffness is close to unity around the minimum of the curve. As *F* increases, the difference in binding affinity for *R* and *D* particles increases making the *R* particles more effective and increasing fiber stiffness.

### Analysis of Fiber Maintenance by the STCM

Both the **TCM** and **STCM** models presented here are able to regulate the fiber stiffness within an interval less than 5% of its initial value over time. However, only the **STCM** can regulate the standard deviation of the stiffness along the fiber. To better understand how the **STCM** achieves this level of control, we performed a simple analytical calculation to shed light on the conditions which must be satisfied in order to obtain a constant *σ*_*k*_. At any instant, the value of each spring constant is the product of the initial value at *t* = 0 and 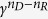, where *n*_*D*_ − *n*_*R*_ is the difference in the number of visits of the two types of particles and *γ* is constant. For many realizations, the distributions of the number of visits, *p*(*n*_*D*_) and *p*(*n*_*R*_), approach a Gaussian distribution at any instant in time. It can be shown that the distribution of *α* = *n*_*D*_ − *n*_*R*_, which determines *k*, also approaches a Gaussian (Supplementary Figs S1 and S2) with a variance given by


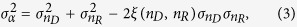


where *ξ* is the correlation function between *n*_*D*_ and *n*_*R*_. Thus, *k* of a single spring is given by *γ*^*α*^*k*_0_, and at each instant in time, the distribution of *k* approaches a lognormal with a variance given by


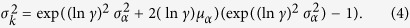


For 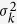 to reach a steady state in the **STCM**, the following two conditions must be met: (*i*) 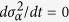 and (*ii*) *dμ*_*α*_/*dt* = 0. Examining Eq. 3, it can be seen that the first condition is satisfied if 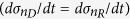 and the correlation function *ξ*(*n*_*D*_, *n*_*R*_) = 1. Condition (*ii*) is equivalent to 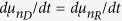, i.e., the two types of particle should visit springs at the same rate.

**Figure 4:**
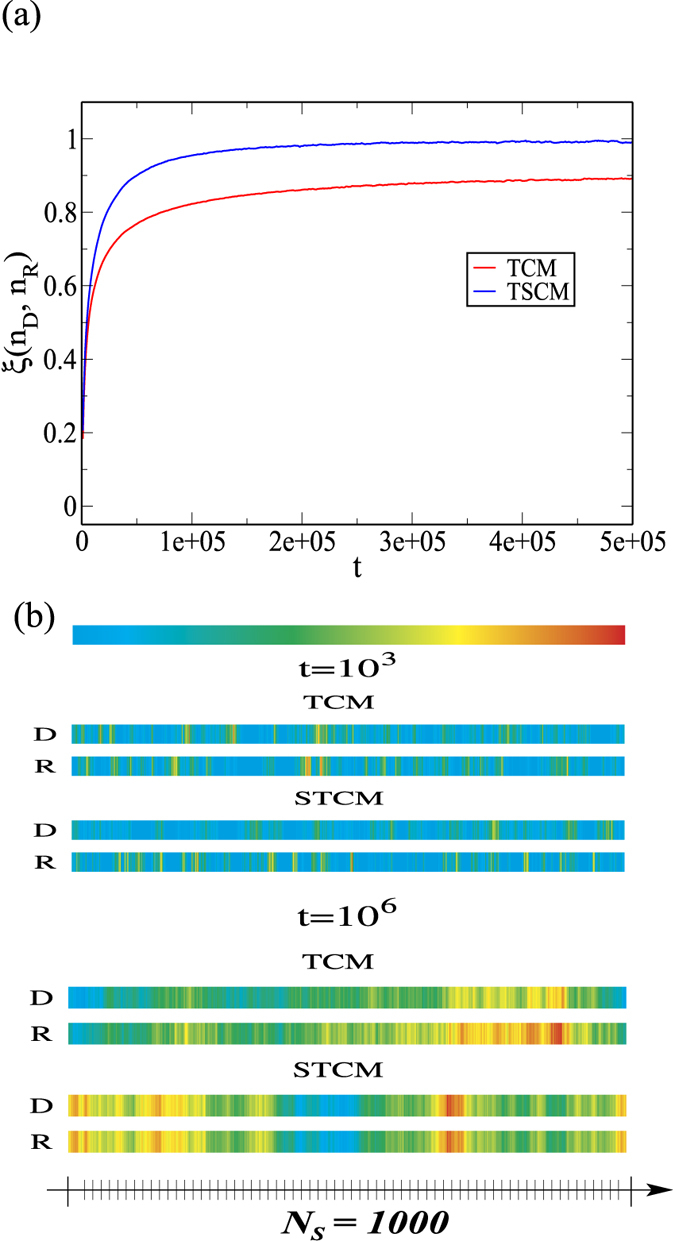
Panel (a) shows the correlation function *ξ*(*n*_*D*_, *n*_*R*_) between the number of visits of *D* and *R* particles as a function of time for the models studied, **TCM** and **STCM**. The correlation function is obtained by computing the correlation coefficient between two corresponding data series, the number of *D* visits along the chain and the number of *R* visits along the chain. Panel (b) presents color maps of the average number of visits, *n*_*D*_ and *n*_*R*_, to every spring along the chain at two time points for the **TCM** and **STCM** models. Blue and red represent low and high number of visits, respectively. The number of visits is normalized by the maximum number of visits at each time point. For the shorter diffusion time of *t* = 10^3^, the color maps for *D* and *R* particle visits are quite different for both models. For the longer time scale of *t* = 10^6^, the color maps for the **TCM** remain different but the **STCM** displays perfectly matched color maps. This means that at each location, the number of visits of the two types of particle is the same balancing local digestion and repair. These results are in agreement with the correlation function that approaches 1 only for the **STCM**. These results were obtained from an average of 500 simulations.

The conditions obtained above are confirmed by the simulation results in Fig. 4a. The correlation function *ξ*(*n*_*R*_, *n*_*D*_) reaches and slightly fluctuates near unity only for the **STCM**. To corroborate that the average number of visits has the same value for both the *D* and *R* particles, we plot in Fig. 4b color maps of the average number of visits per spring for both particles along the chain for short and long time scales, *t* = 10^3^ and *t* = 10^6^. The color maps are identical for both types of particles at longer time points, which is in agreement with the calculations in the case of **STCM**. Thus, the **STCM** as a model of biological regulation of fiber maintenance fulfills the requirement to keep both elasticity and homogeneity of a fiber within a narrow range in the presence of continuous degradation and repair. This can be achieved only in the presence of tension on the fiber which places part of the regulatory mechanism outside the cell because the binding affinity becomes a function of the applied force as well as the local stiffness along the fiber.

### Comparison of Model Prediction to Experimental data

Comparison of our results with experimental data would require measurements of the variation of the Young’s modulus of individual collagen fibrils along its axis. To our knowledge, no such Young’s modulus measurements in the axial direction are available along the collagen fibers. Atomic Force Microscopy measurements along the fibril in indentation mode showed significant axial heterogeneity of stiffness^26^; however, the indentation induces both compression and shear and since collagen is anisotropic with different axial and lateral moduli, such data are not directly applicable to estimate the Young’s modulus in axial extension. Nevertheless, a positive correlation between average fiber diameter and the low strain modulus has been found for artificial assemblies of type I collagen fibers^27^. To extend this to a relation between local diameter and local stiffness, we note that the stiffness at any given location should be related to the number of collagen monomers *N*_*c*_ that pass the cross section *A* of the fiber. If we assume that the cross-link density is constant along the fiber, the local stiffness should be proportional to *N*_*c*_. Since *A* is the sum of the cross sectional areas of individual monomers, *A* is also proportional to *N*_*c*_ implying that fiber diameter is proportional to the square root of *N*_*c*_. Thus, we compute the distribution of *k*^1/2^ and compare it with the distribution of diameters along individual collagen fibrils.

**Figure 5:**
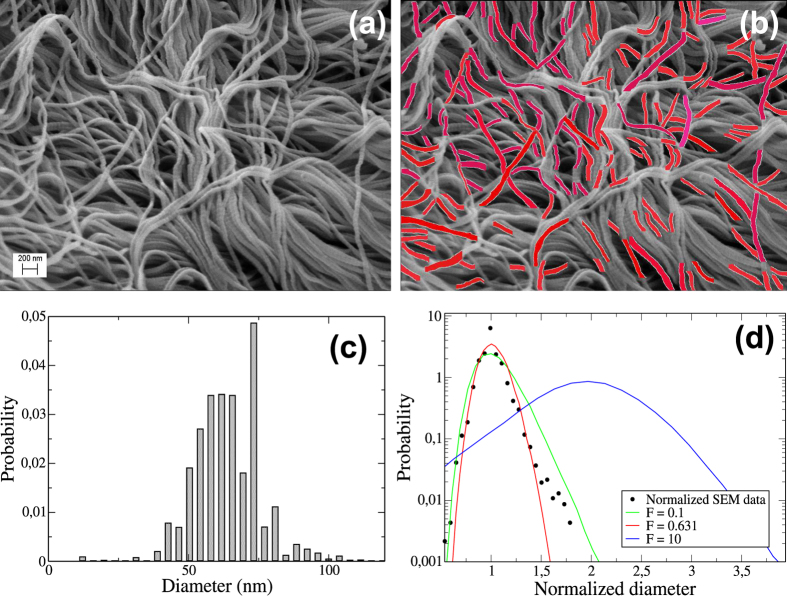
Panel (a) shows part of the collagen fiber structure of the thoracic aorta of a healthy adult rat obtained using scanning electron microscopy. The highlighted parts in (**b**) are the segments selected for measurement of diameters along the axis of fibers. (**c**) presents the distribution of diameters exhibiting a bi-modal shape. In (**d**) the diameters corresponding to each fiber are normalized by the median of the fiber (filled black circles). Note that the distribution now shows a single peak. Also shown are several model simulations of predicted diameter distributions corresponding to 3 values of *F* in the **STCM**. Note that for *F* = 0.6, the simulated distribution matches the experimental one.

Images of the thoracic aorta of normal rats were obtained by scanning electronic microscopy (Fig. 5a). After image processing, segments were selected if their borders could be identified properly using an edge detection method. A set of 165 segments were included in the final analysis. The results of this process are shown in Fig. 5b. The distribution of diameters exhibits two peaks (Fig. 5c) similar to recent results obtained by measuring cross sectional areas on images perpendicular to the fiber axis^8^. We next normalize each diameter value along a fiber with the corresponding median diameter so as to obtain comparable variations along fibers for all fibers. The corresponding distribution is compared to the distributions of *k*^1/2^ for 3 values of *F* in Fig. 5d. We find a good match between our experimental results and the model simulations with *F* = 0.6, which corresponds to the minimum of *σ*_*s*_ in Fig. 3b or the maximum homogeneity of the fiber. Note that no formal fitting of the model was carried out to fit the data. Also, both the experimental and the model predicted diameter distributions are nearly Gaussian with a narrow variability of 13% around the mean.

## Discussion

In this study, we investigated the cellular long-term maintenance of an elastic fiber under tension including degradation and repair processes combined with diffusion of regenerative and degradative particles by various computational models. Our main finding is that a homeostatic fiber stiffness can be achieved easily by assuming that cells periodically probe the overall stiffness of the fiber and properly adjust the production and release of degradative enzymes and regenerative monomers. However, this control mechanism fails to maintain a homogeneous fiber that can be seen on images of collagenous tissues. We also introduced the idea that part of the control mechanism is locally governed by how the biophysical properties of the fibers including binding affinity are modulated by the applied force on the fiber. This model predicts diameter variations along the fiber that are in quantitative agreement with experimental data when the applied force is in the range where the variance of local stiffness is at its minimum. Thus, our model predicts a strong involvement of the fibers in their own repair that warrants further experimental studies. While little is known about how tensile forces regulate fiber repair^16^, more studies reported regulation of ECM degradation by mechanical forces^15^,^17^,^25^,^28^,^29^. The combined regulation of both repair and digestion by mechanical forces can be studied computationally; however, it needs to be tested experimentally how the on rate of the regenerative particles changes with mechanical forces on the fibers.

Various computational models have been developed to describe tissue remodeling and homeostasis^30^,^31^,^32^. However, to our knowledge, our model is unique in that it investigates the combined effects of diffusion and binding of two different particles on the long-term homeostatic structure and function of fibers. An interesting result of the simulations is that cellular control of long-term fiber maintenance is possible only when tension is applied to the fibers, which in turn predicts the possibility of a homeostatic optimal condition for the fiber. Indeed, as Fig. 3 demonstrates, both fiber structure characterized by the axial variations of diameter and fiber function characterized by the global stiffness can be regulated by mechanical forces to achieve a minimum value. Furthermore, these fiber properties are not independent of each other as they are both a consequence of the axial distribution of stiffness. This represent an important structure-function relation for the fiber which may offer a way to control fiber and tissue mechanical properties through the different pre-existing mechanical stresses in various organs. Indeed, the transpulmonary pressure in the lung is between 0.5 and 1 kPa^33^ whereas the transmural pressure in arteries is around 10–15 kPa^34^. This is another prediction of the model that can be tested experimentally.

Our model also has limitations. First, the model is 2-dimensional while the diffusion of particles in the ECM occurs in 3 dimensions. We tested the influence of adding 5 layers in a star-shape configuration (Supplementary Fig. S3a) in which the diffusing particle can step left, right or onto the fiber, but not directly from one layer to another. However, when the particle unbinds, it can step onto any of the five layers. We find that the distribution of *α* is the same in the 2-layer and 5-layer models. We also test another model, called the ring configuration (Supplementary Fig. S3b), in which the diffusing particle is allowed to step left, right, onto the fiber as well as across the layers. Again, the number of layers does not matter; however, in this case, the distributions of *α* as well as *σ*_*k*_ are different from those in the star configuration (Supplementary Fig. S4). The reason is that in the ring configuration, the diffusing particle can spend more time around a single binding site and this is similar to adding a waiting time to the diffusion that changes the axial diffusion constant. Consequently, the particle can step onto the same binding site more often and generate stronger heterogeneity of *k* along the fiber. Another limitation is that two particles can step on the same site during diffusion. When we include volume exclusion not only on the fiber but also on the diffusion sites, the dynamics of the control becomes slightly slower because the number of visits at a given site is slightly reduced (Supplementary Figs S5 and S6). The introduction of volume exclusion also leads to the number of diffusion layers becoming a small factor in the steady state of the variance (Supplementary Fig. S7). With respect to the periodic assessment of fiber stiffness we note the following. The fraction *f* of particles relabeled as *R* or *D* affects the average stiffness (Fig. 3c) showing a minimum around *F* = 0.6, but not the steady state numbers of *R* and *D* particles around the fiber which are determined by the on and off rates (Supplementary Figs S8 and S9). Thus, *f* can be considered as the strength of cellular control and hence it represents an effective turnover rate of collagen.

To investigate biologically more realistic scenarios, we first incorporate slower diffusion and more “stickyness” for the *R* particles which represent a relatively large protein, the collagen monomer, compared to the smaller enzymes. The effect of this modification is to slow down the control (not shown). Finally, to account for the observed experimental fact that the enzyme carries out a biased random walk moving essentially in one direction along the fiber^35^, we introduce a multiplicative factor to make diffusion stepping in one direction *B* times more likely than in the other direction. Interestingly, this reduces *σ*_*k*_ in the steady state because the enzyme spends less time at a binding site that may not need cleaving. Supplementary Fig. S10 compares this model with the original for different values of *B* suggesting that the efficiency of the control mechanism can be improved by specific features of the interaction of the collagen and its degradative enzymes. For larger and more mature fibers, cleaving or adding a monomer should have less of an effect. We test this by examining the sensitivity of stiffness and variance to *γ*. As the value of *γ* approaches unity, the fiber stiffness also approaches unity and *σ*_*k*_ decreases (Supplementary Fig. S11). Finally, we note that the choice of assigning tension dependence to the *R* particles is related to contradictory results about how tension affects enzyme kinetics. While several studies suggested that tension protects against cleavage^15^,^28^, one study reported that tension accelerates digestion^29^. The choice of which particle binding is sensitive to tension should not reduce the efficiency of the control mechanism. Indeed, if the probability of binding of *D* particles to the fiber is more efficient in regions where the local stretch is low, full stability can easily be achieved (Supplementary Fig. S12). Thus, while the effective stiffness and homogeneity of the fiber can be regulated by tuning various parameters, none of the above details affect the ability of the control mechanism to achieve a robust steady state.

To conclude, our model provides new insight into the long-term regulation of ECM fibers. Additionally, the model can be used to study the progression of diseases in which the long-term cellular maintenance of the ECM is disturbed such as in fibrosis, pulmonary emphysema or cancer. Our results may also have implications for aging of the ECM. During aging, non-enzymatic cross-linking of collagen steadily progresses and this process likely hinders the cellular assessment of stiffness and/or alters the tension dependent biophysical properties of fibers so that the active involvement of the fibers in their own maintenance is less functional. Finally, the concept developed here may also advance the field of tissue engineering by contributing to the design of biomaterials with self-repairing ability.

## Methods

### Numerical Calculations

In all numerical simulations described in the main text, the chain was formed by *N*_*s*_ = 1000 springs in series. Except for the simulations with no periodic regulation, the total number of particles was 100. At the beginning, the particle types (*D* or *R*) were chosen randomly before the periodic relabeling process took place. At each time step, all 100 particles were allowed to move sequentially according to the rules described in the main text. In all simulations the averages were taken over 500 and occasionally 1000 realizations, which we verified to produce adequate convergence.

### Scanning Electron Microscopy

All procedures were approved by the Institutional Animal Care and Use Committee of Boston University (Protocol # 12-031) and the experiments were performed in accordance with relevant guidelines and regulations. Rats (*n* = 3) were sacrificed and the thoracic aorta was isolated as described previously^36^. Samples were fixed in a mixture of 3% glutaraldehyd 3% paraformaldehyde in 0.1 M cacodylate buffer, pH 7.4 at 4 °C for 24 hours, washed 6x in 0.1 M cacodylate buffer pH 7.4, then gradually dehydrated in an ethanol series (1 × 10 min. in 25% ethanol, 1 × 10 min. in 50% ethanol, 1 × 10 min. in 70% ethanol, 1 × 10 min. in 85% ethanol, 1 × 10 min. in 95% ethanol, 2 × 10 min. in 100% ethanol, 1 × 10 min. in 100% ethanol (EM grade). The samples were then critical point dried, and gold/palladium sputter coated. Imaging was done in a Zeiss Supra 55VP Field Emission Scanning Electron Microscope using electron beam energy of 5 kV.

### Image Processing

We first applied the Canny edge detection algorithm and compared the resulting images with their originals. This allowed us to manually remove those segments from the processed image that did not correspond to true fiber boundaries. For diameter measurements, we selected a total of 165 fiber fragments from the segmented images. The mid-line of each segment was found by applying an image transformation that determined, for every pixel within the pair of fiber boundaries, the distance to the nearest pixel outside the borders. This resulted in 8138 distance values for the diameters of the segments. The diameter values along each fiber were then normalized by the median diameter of the segment.

## Additional Information

**How to cite this article**: Alves, C. *et al*. Homeostatic maintenance via degradation and repair of elastic fibers under tension. *Sci. Rep.* **6**, 27474; doi: 10.1038/srep27474 (2016).

## Supplementary Material

Supplementary Information

## Figures and Tables

**Table 1:** Summary of model parameters and measured quantities.

*K*(*t*)/*K*_0_	Normalized stiffness of the fiber at instant *t*
*σ*_*k*_	Standard deviation of local spring stiffnesses
〈*k*_*s*_〉	Mean of spring stiffnesses in the steady state
*σ*_*s*_	Standard deviation of spring stiffnesses in the steady state
*N*_*D*_, *N*_*R*_	Number of *D* and *R* particles in the system. The sum *N*_*D*_ + *N*_*R*_ = 100 is kept constant.
*t*_*u*_	Time period of updating particle types
*f*	Fraction of particles relabeled at each update event
*F*	Force applied to the fiber per unit of length
*p*_*on*,*D*_, *p*_*on*,*R*_	probabilities of binding for *D* and *R* particles. While *p*_*on*,*D*_ = 1/3 is kept constant, *p*_*on*,*R*_ depends on *F* (Eq. 2).
*p*_*off*_	probability of unbinding
