# Supplementary material for: Homeostatic maintenance via degradation and repair of elastic fibers under tension
Source: Sci Rep. 2016 Jun 9;6:27474. doi: 10.1038/srep27474 (PMC4899696; doi:10.1038/srep27474)
Supplement: Supplementary Information [file srep27474-s1.pdf]

Supplementary Information

Homeostatic maintenance via degradation and repair of elastic fibers under tension

Calebe Alves[1,2], Ascanio D. Araújo[1], Cláudio L. N. Oliveira[1], Jasmin Imsirovic[2], Erzsébet Bartolák-Suki[2], José S. Andrade[1] and Béla Suki[2,*]

[1]Universidade Federal do Ceará, Departamento de Física, Fortaleza, CE, 60451-970, Brazil

[2]Boston University, Department of Biomedical Engineering, Boston, MA, 02215, USA

[*]bsuki@bu.edu

April 13, 2016

Number of visits to binding sites

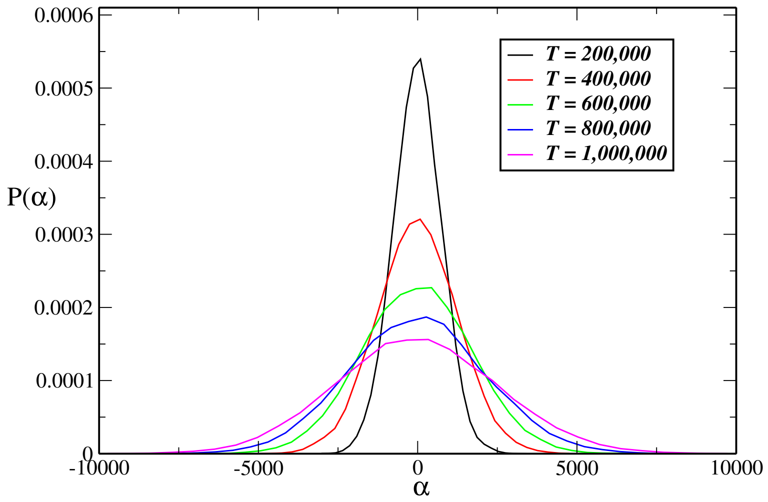

Figure S1: Distribution of α_i at different times (statistics accumulated over 500 realizations), in the model without control.

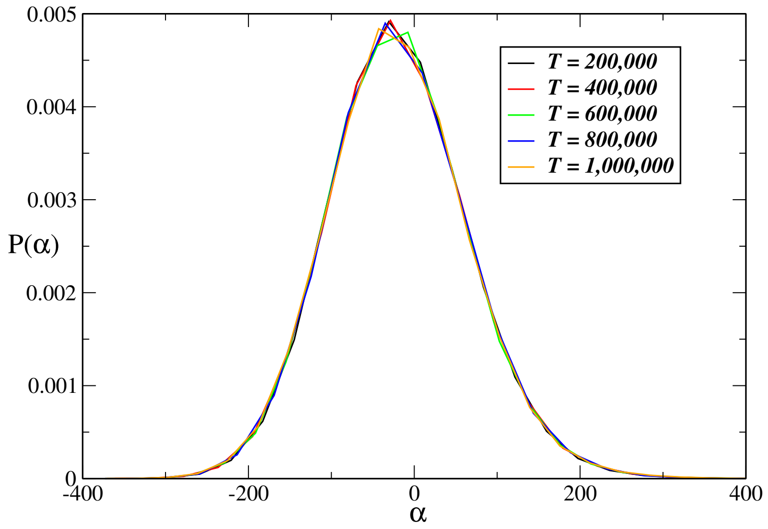

Figure S2: Distribution of α_i at different times (statistics accumulated over 500 realizations), in the STCM.

Different number of Diffusion Site Layers

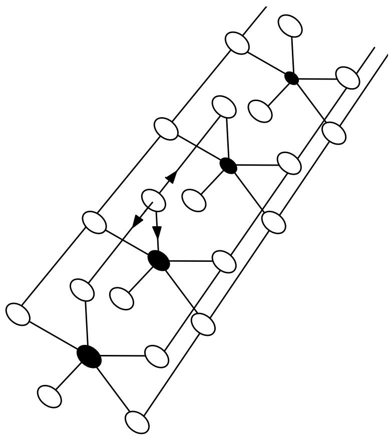

(a) The "star" geometry.

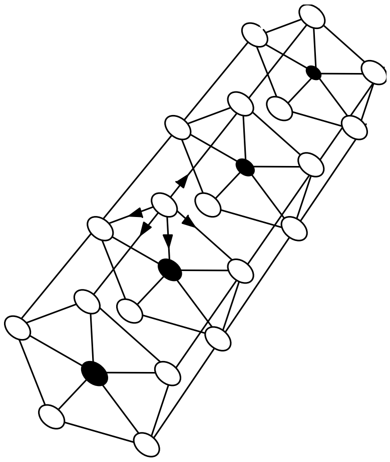

(b) The "ring" geometry.

Figure S3: Considered geometries for the fiber.

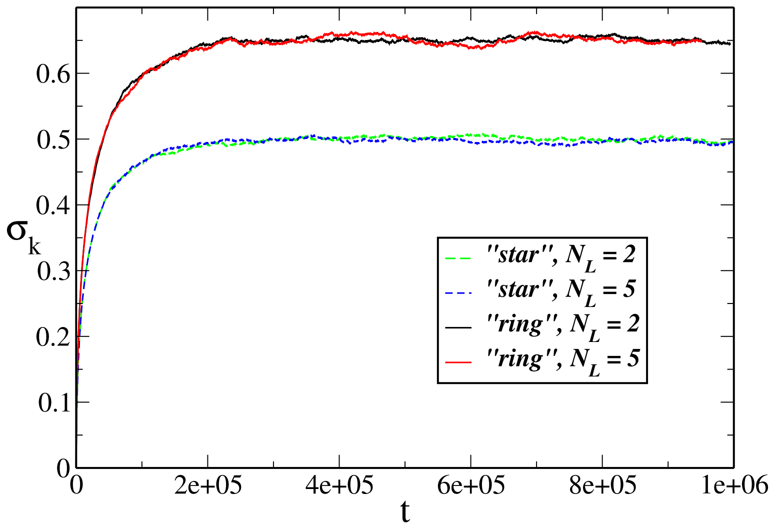

Figure S4: Effect of Different number of layers in the STCM model.

Effects of Volume Exclusion

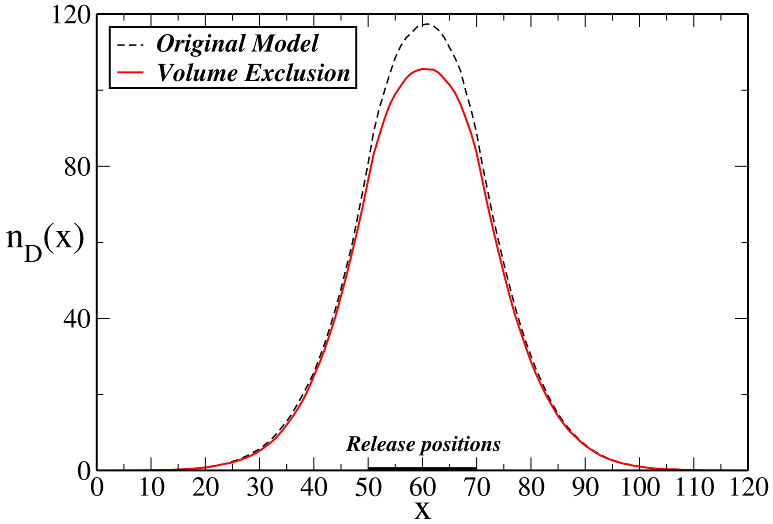

Figure S5: Average number of visits to site x, when there's no control and 20 particles are released on the central sites as shown, after $T = 10^3$ timesteps. These central sites are less visited than they are in original model. Distributions obtained from $40,000$ realizations in the model without control.

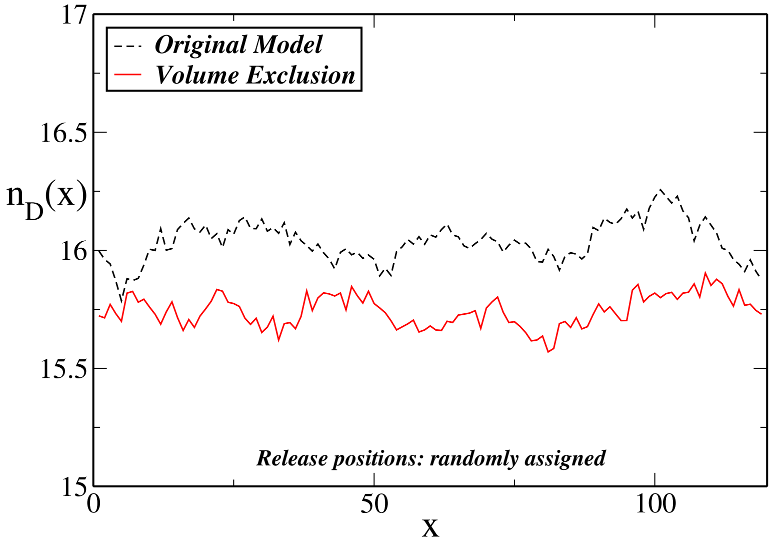

Figure S6: Average number of visits to site x, when there's no control and particles are released at random positions, after $T = 10^3$ timesteps. All sites are less visited than they are in original model. Averages obtained from 40,000 realizations in the model without control.

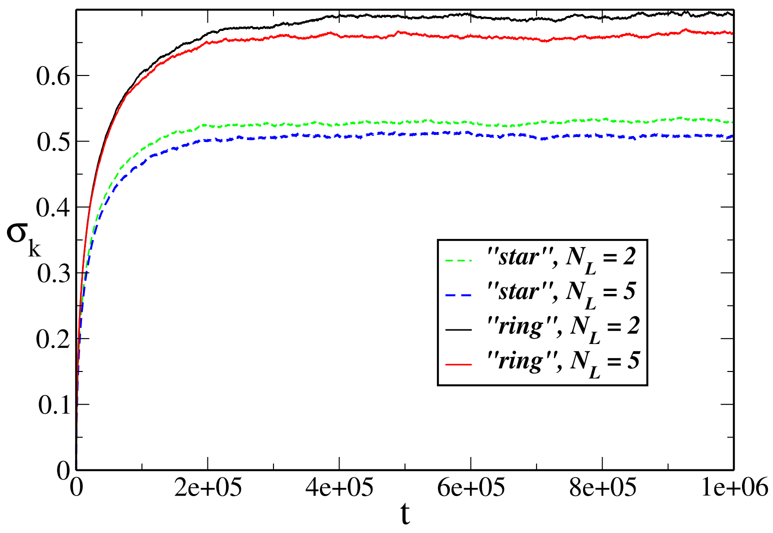

Figure S7: Effect of Volume Exclusion on σ_k, for different geometries and number of layers in the STCM.

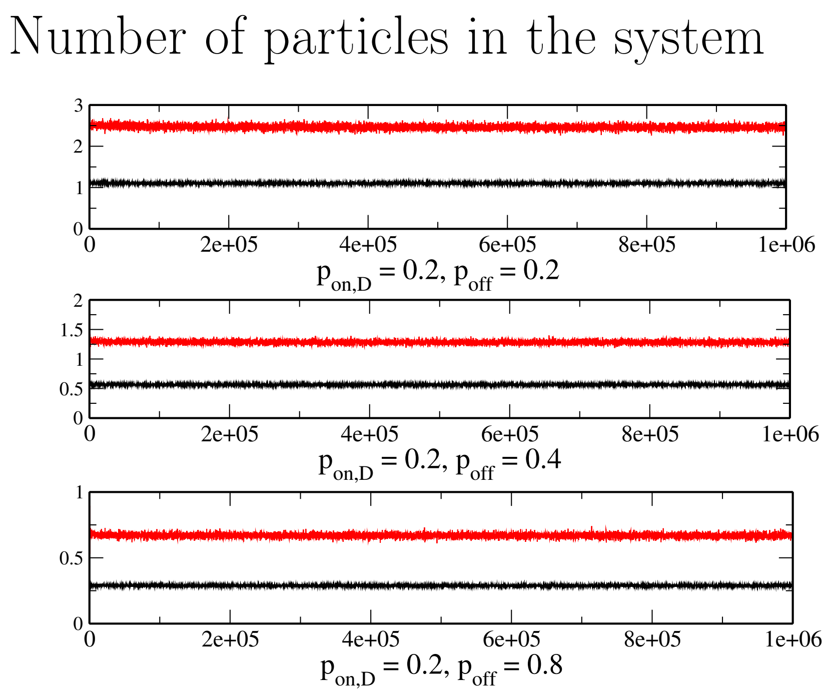

Figure S8: Influence of p_{off} and p_{on} on the number of bound and free particles. Black: ratio between bound D particles and free D particles as a function of time; Red: ratio between bound R particles and free R particles. Parameters used in the STCM are: $F = 1.0$, $t_u = 100$, $f = 0.3$.

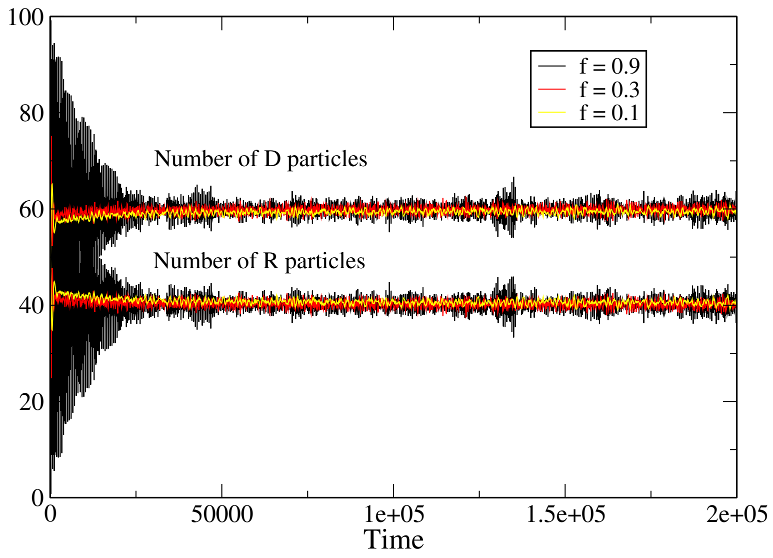

Figure S9: Infuence of the parameter f on the STCM.

Effect of Biased Diffusion

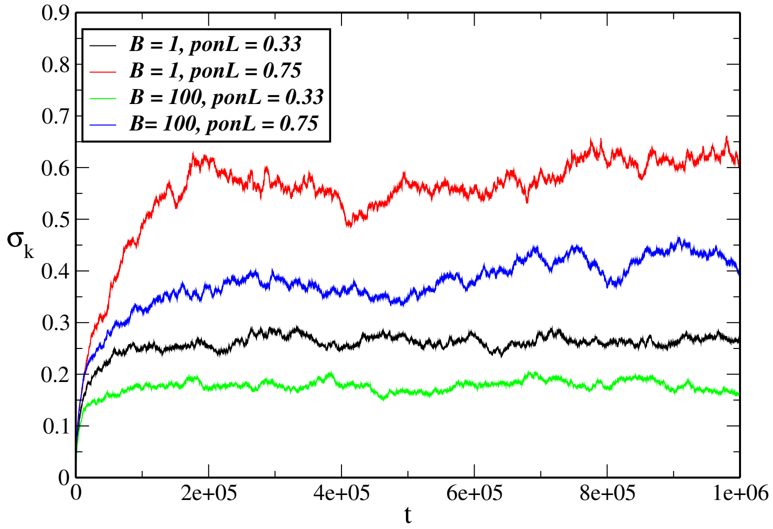

Figure S10: When particles move preferentially in one direction, the control mechanism becomes more efficient. The ratio of probabilities of stepping to the right and to the left is B. The probability of unbinding for R particles is $p_{on,R} = 1 - (1 - p_{on,L} \exp(-F/k))$ so that $p_{on,L}$ is the probability to bind to a spring with large stiffness.

Effect of parameter γ

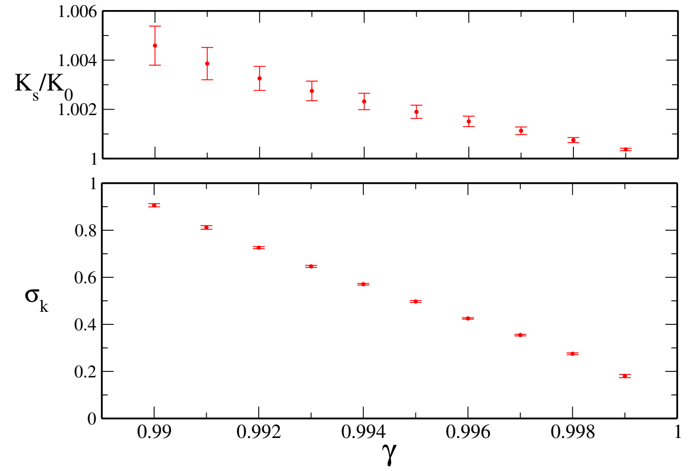

Figure S11: Normalized fiber stiffness K_s and Standard deviation of spring stiffnesses σ_s in the steady state as a function of γ in the STCM. Parameters used were $F = 1.0$, $t_u = 100$ and $f = 0.3$.

Effect of F on the on rate of D particles

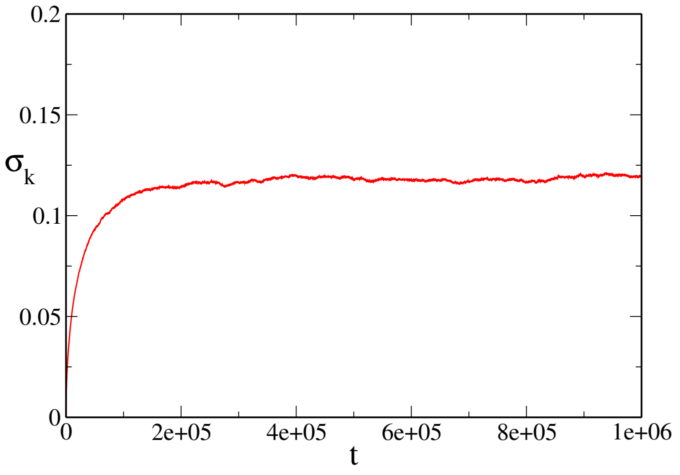

Figure S12: Standard deviation of spring stiffnesses when D particles are sensitive to tension, but R particles are not. Here, the probability for a D particle to bind to a spring with stiffness k is $p_{on,D} = \frac{1}{1+F/k}$. For R particles, the probability was kept constant and equal to $p_{on,R} = \frac{1}{3}$. Parameters used are $F = 1.0$, $t_u = 10$ and $f = 0.3$.
